# On the direct insulator-quantum Hall transition in two-dimensional electron systems in the vicinity of nanoscaled scatterers

**DOI:** 10.1186/1556-276X-6-131

**Published:** 2011-02-11

**Authors:** Chi-Te Liang, Li-Hung Lin, Chen Kuang Yoa, Shun-Tsung Lo, Yi-Ting Wang, Dong-Sheng Lou, Gil-Ho Kim, Chang Yuan-Huei, Yuichi Ochiai, Nobuyuki Aoki, Jeng-Chung Chen, Yiping Lin, Huang Chun-Feng, Sheng-Di Lin, David A Ritchie

**Affiliations:** 1 Department of Physics, National Taiwan University, Taipei 106, Taiwan; 2 Department of Applied Physics, National Chiayi University, Chiayi 600, Taiwan; 3 Department of Physics, National Tsinghwa University, Hsinchu 300, Taiwan; 4 Department of Electronic and Electrical Engineering and SAINT, Sungkyunkwan University, Suwon 440-746, Korea; 5 Graduate School of Advanced Integration Science, Chiba University, Chiba 263-8522, Japan; 6 National Measurement Laboratory, Centre for Measurement Standards, Industrial Technology Research Institute, Hsinchu 300, Taiwan; 7 Department of Electronics Engineering, National Chiao Tung University, Hsinchu 300, Taiwan; 8 Cavendish Laboratory, J.J. Thomson Avenue, Cambridge CB3 0HE, UK

## Abstract

A direct insulator-quantum Hall (I-QH) transition corresponds to a crossover/transition from the insulating regime to a high Landau level filling factor ν > 2 QH state. Such a transition has been attracting a great deal of both experimental and theoretical interests. In this study, we present three different two-dimensional electron systems (2DESs) which are in the vicinity of nanoscaled scatterers. All these three devices exhibit a direct I-QH transition, and the transport properties under different nanaoscaled scatterers are discussed.

## Introduction

The simultaneous presence of disorder and a strong enough magnetic field *B *can lead to a wide variety of interesting physical phenomena. For example, the integer quantum Hall effect is one of the most exciting effects in two-dimensional electron systems (2DES), in which the electrons are usually confined in layers of the nanoscale [1]. In an integer quantum Hall (QH) state, the current is carried by the one-dimensional edge channels because of the localization effects. It has been shown that with sufficient amount of disorder, a 2DES can undergo a *B*-induced insulator to quantum Hall transition [2-5]. Experimental evidence for such an insulator-quantum Hall (I-QH) transition is an approximately temperature (*T*)-independent point in the measured longitudinal resistivity of a 2DES [3-5]. The I-QH transition continues to attract a great deal of interest both experimentally and theoretically as it may shed light on the fate of extended states [6-10], the true ground state of a non-interacting 2DES [2], and a possible metal-insulator transition in 2D [11,12].

It is worth pointing out that in order to observe an I-QH transition separating the zero-field insulator from the QH liquid, one needs to deliberately introduce strong disorder within a 2DES. The reason for this is that the localization length needs to be shorter than the sample size. In the study by Jiang and co-workers [2], a 2DES without a spacer layer in which strong Coulomb scattering exists was used. Wang et al. utilized a 30-nm-thick heavily doped GaAs layer so as to allow the positively charged Si atoms to introduce long-range random potential in the 2DES [3]. Hughes et al. have shown that when a Si-doped plane was incorporated into a 550-nm-thick GaAs film, a deep potential well can form in which the 2DES is confined close to the ionized donors and is therefore highly disordered [4]. It has been shown that by deliberately introducing nanoscaled InAs quantum dots [13] in the vicinity of a modulation-doped GaAs/AlGaAs heterostructure, a strongly disordered 2DES which shows an I-QH transition can be experimentally realized [14,15].

The transition/crossover from an insulator to a QH state of the filling factor ν > 2 in an ideal spinless 2DES can be denoted as the direct I-QH transition [16-19]. Such a transition has been attracting a great deal of interest and remains an unsettled issue. Experimental [16-19] and theoretical results [9,10] suggest that such a direct transition can occur, and it is a quantum phase transition. However, Huckestein [20] has argued that such a direct transition is not a quantum phase transition, but a narrow crossover in *B *due to weak localization to Landau quantization.

In this study, the authors compare three different electron systems containing nanoscaled scatterers which all show a direct I-QH transition. The first sample is a GaAs 2DES containing self-assembled nanoscaled InAs quantum dots [13,14,21-23].

The second one is a 2DES in a nominally undoped AlGaN/GaN heterostructure [24-33] grown on Si substrate [33,34]. Such a GaN-based electron system can be affected by nanoscaled dislocation and impurities [35]. Finally, experimental results on the third sample, a delta-doped GaAs/AlGaAs quantum well with additional modulation doping [36,37], will be presented. All the experimental results on the three completely different samples show that the direct I-QH transition does not occur with the onset of strong localization due to Landau quantization [20,38]. Therefore, in order to obtain a thorough understanding of the direct I-QH transition, further studies are required.

### Experimental details

**Figure 1:**
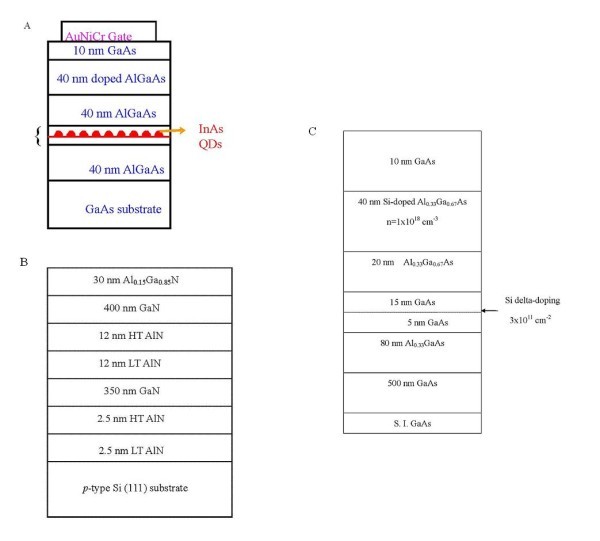
**Schematic diagrams showing the structure of (a) Sample A, (b) Sample B, and (c) Sample C**.

Figure 1a,b,c show the structures of the three devices, Sample A, Sample B, and Sample C, considered in this study. Sample A is a GaAs/AlGaAs 2DES containing self-assembled InAs quantum dots. Sample B is an AlGaN/GaN heterostructure grown on Si. Such a system is fully compatible with Si CMOS technology and is thus of great potential applications. Sample C is a delta-doped quantum well with additional delta-doping. Since the electrons in the quantum well in sample B are in close proximity of nanoscaled dislocation and impurities, the 2DES is strongly influenced by these nanoscaled scatterers. In fact, these scatterers provide scattering which is required for observing the I-QH transition [16]. On the other hand, the scatterings in samples A and C are mainly due to the self-assembled quantum dots and the delta-doping in the quantum well, respectively. Recent studies focussing on alloy disorder in Al_x_Ga_1-x_As/GaAs heterostructure [39-41] have shown that 2DESs influenced by short-range disorder provides an excellent opportunity to connect the Anderson localization theory with real experimental systems [41]. Moreover, the nature of disorder may affect scaling behavior in the plateau-plateau (P-P) transition at high *B *[39-41], and the P-P and I-QH transitions may be considered as the same universality class [42]. Therefore, it may be interesting to investigate the direct I-QH transitions under different scattering types at low magnetic fields. In this article, such low-field direct transitions in samples A, B, and C are compared.

**Figure 2:**
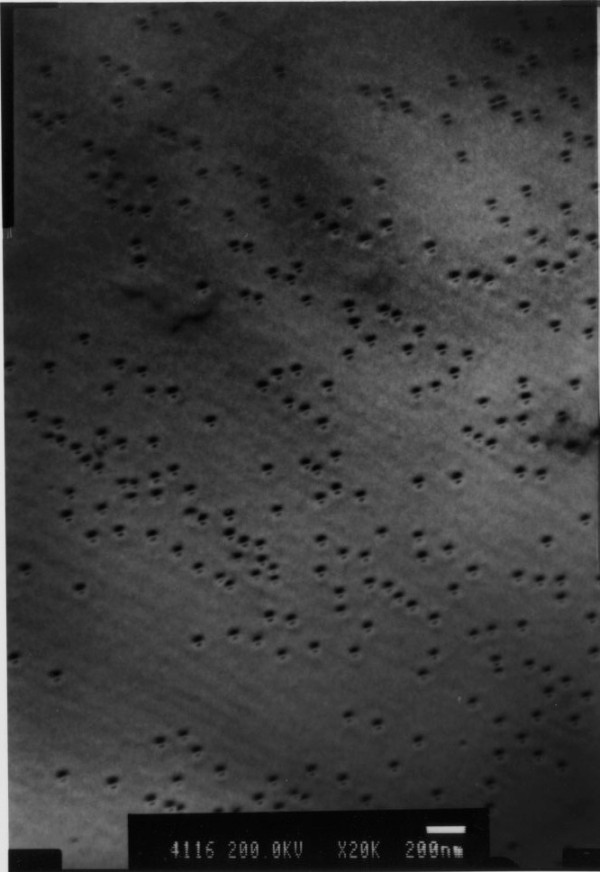
**A plane-view of TEM image of the wafer which was cut to fabricate sample A**.

Figure 2 shows a TEM image of the wafer for fabricating Sample A. Very uniform nanoscaled InAs quantum dots can be seen. These nano-scattering centers provide strong scattering in the vicinity of the 2DES in the GaAs. The dimensions of the quantum dot are estimated to be 20 nm in diameter and 4 nm in height. Experiments were performed in a top-loading He3 cryostat equipped with a superconductor magnet. Four-terminal resistance measurements were performed using standard phase-sensitive lock-in techniques.

## Results and discussions

**Figure 3:**
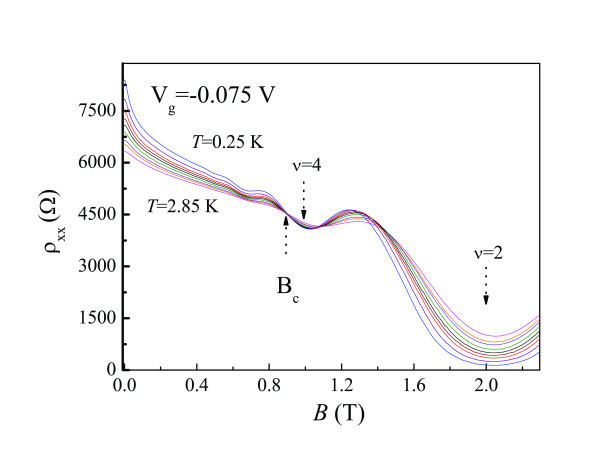
**ρ_xx_(*B*) at various temperatures ranging from 0.25 to 2.85 K (Sample A)**.

Figure 3 shows the longitudinal magnetoresistivity measurements on Sample A as a function of *B *at various temperatures. It can be seen that at a crossing field *B*c = 0.9 T, ρ_xx _is approximately *T*-independent. For *B *<*B*_c_, ρ*_xx_*decreases with increasing temperature, characteristics of an insulating regime [16]. For *B *>*B*_c_, ρ*_xx_*increases with increasing temperature, and therefore the 2DES is in the quantum Hall regime. As the 2DES enters the ν = 4 QH state from the insulating regime, a direct 0-4 transition where the symbol 0 corresponds to the insulator has been observed. It is worth pointing out that before the 2DES enters the ν = 4 QH state, resistance oscillations due to Landau quantization in the insulating regime have already been observed [15,19,21]. Therefore, the experimental results of this study clearly demonstrate that the crossover from localization from Landau quantization actually covers a wide range of magnetic field, in sharp contrast to Huckestein's argument [19-21].

**Figure 4:**
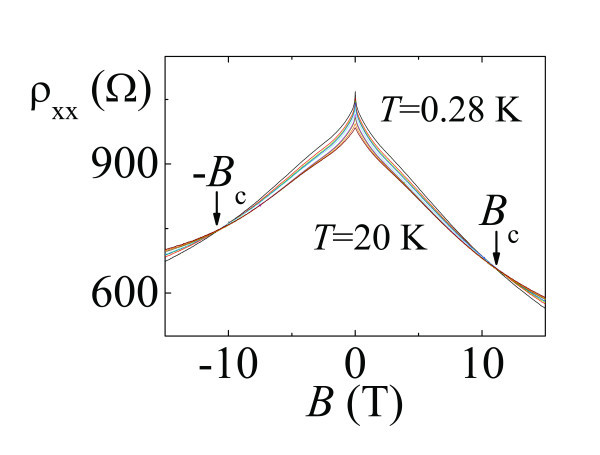
**ρ_xx_(*B*) at various temperatures ranging from 0.28 to 20 K (Sample B)**.

As mentioned earlier, a GaN-based electron system can be affected by nanoscaled dislocation and impurities. It is therefore interesting to study such a system. Figure 4 shows magnetoresistance measurements on Sample B as a function of magnetic field at different temperatures. The data deviate slightly from the expected symmetric behavior, i.e., *R*(*B*) = *R*(-*B*). The reason for this could be due to slight misalignment of the voltage probes. Nevertheless, it can be seen that at *B*_c_ = 11 Tand -*B*_c_ = -11 T, the measured resistances are approximately temperature independent. The corresponding Landau level filling factor is about 50 in this case. Therefore, a direct 0-50 transition has been observed. Note that even at the highest attainable field of approximately 15 *T*, there is no sign of resistance oscillations due to the moderate mobility of our GaN system. Therefore, the experimental results of this study clearly demonstrate that the observed direct I-QH transition is irrelevant to Landau quantization. Therefore, the onset of Landau quantization does not necessarily accompany the direct I-QH transition, inconsistent with Huckestein's model [20].

**Figure 5:**
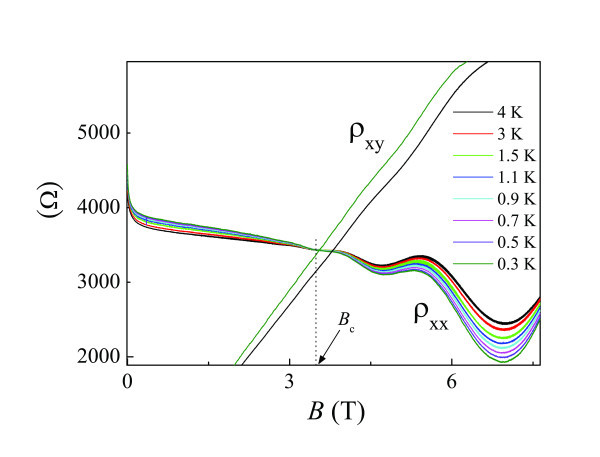
**ρ_xx_(*B*) at various temperatures ranging from 0.3 to 4 K (Sample C)**. ρ*xx *at *T *= 0.3 K and *T *= 4 K are shown.

**Figure 6:**
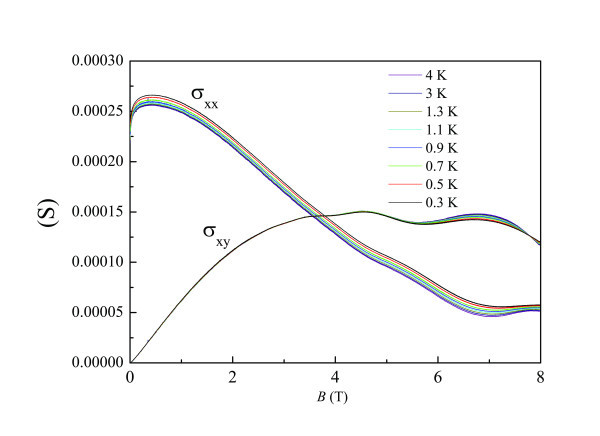
**Converted σ_xx_(*B*) and σ_xy_(*B*) at various temperatures ranging from 0.3 to 4 K (Sample C)**.

Figure 5 shows magnetoresistance measurements on Sample C as a function of magnetic field at various temperatures. It can be seen that the 2DES undergoes a 0-8 transition characterized by an approximately temperature-independent point in ρ *_xx_*at the crossing field *B*_c_. Near the crossing field, ρ*_xx_*is very close to ρ*_xy_*though ρ*_xy_*shows a weak *T *dependence. For *B *<*B*_c_, no resistance oscillation is observed. At first glance, our experimental results are consistent with Huckestein's model. However, it is noted that Landau quantization should be linked with quantum mobility, not classical Drude mobility [36]. Moreover, the observed oscillations for *B *>*B*_c_ do not always correspond to formation of quantum Hall states. As mentioned in our previous study [36], the observed oscillations can be well approximated by conventional Shubnikov-de Haas (SdH) formalism. It is noted that the SdH formula is derived without considering quantum localization effects which give rise to formation of quantum Hall state. Therefore, quantum localization effects are not significant in the system under this study. Actually, as shown in Figure 6, the crossing point in σ*_xy_* at around 7.9 Tmay correspond to the extended states due to the onset of the strong localization effects. Therefore, in this study, the onset of strong localization actually occurs at a magnetic field approximately 4 Thigher than the crossing point.

It has been suggested that by converting the measured resistivities into longitudinal and Hall conductivities, it is possible to shed more light on the observed I-QH transition [5]. Figure 6 shows such results at various temperatures. Interestingly, for *B *< 5 T, _σ_*_xy_*is nominally *T *independent. Such data are consistent with electron-electron interaction effects. Over the whole measurement range, σ*_xx_*decreases with increasing *T*, consistent with electron-electron interaction effects. Unlike σ_*xy*_, σ*_xx_*shows a significant Tdependence.

By inspecting the conductivies, previously the authors have studied the renormalized mobility [43] of a GaN-based 2DES at high temperatures (Sample B) [44]. It is therefore interesting to study such a mobility for both Sample A and Sample C. It has been suggested the electron-electron interaction effects can renormalize the mobility μ' given by

(1) $$\sigma_{xy}=\frac{ne\mu {'}^{2}B}{1+{\left(\mu 'B\right)}^{2}},$$

(2) $$\sigma_{xx}=\frac{ne\mu 'B}{1+{\left(\mu 'B\right)}^{2}}+\Delta \sigma_{ee}^{d}.$$

**Figure 7:**
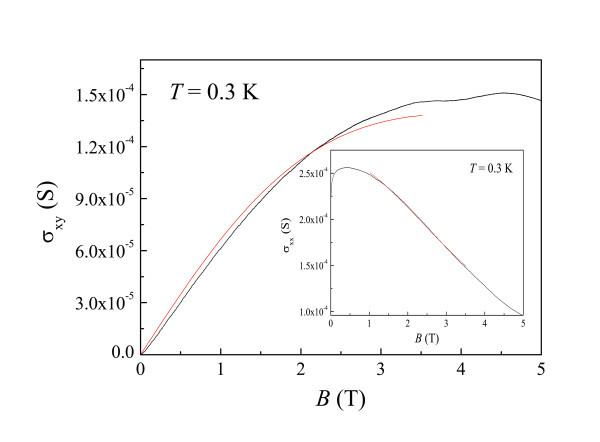
**σ_xy_(*B*) and the fit to Equation 1 for 0 <*B *< 3.5 T**. The inset shows σ_xx_(*B*) and the fit to Equation (2) for 1 T <*B *< 3.5 T.

**Figure 8:**
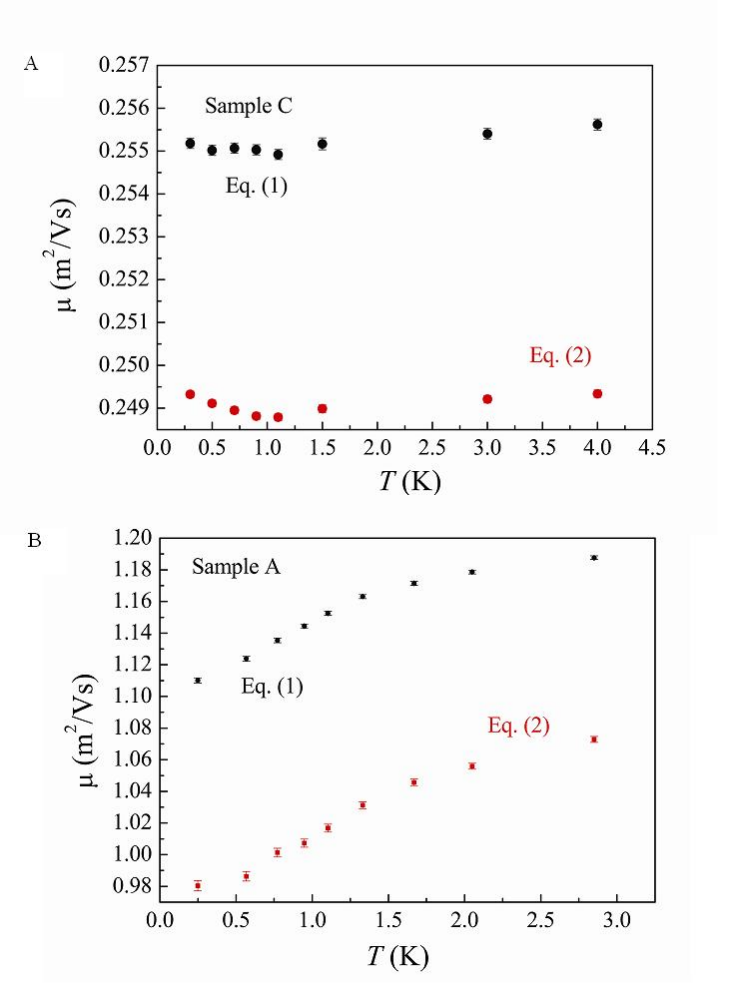
**Calculated renormalized mobilities due to electron-electron interaction effects using Equations (1) and (2) for (a) Sample C and (b) Sample A, respectively**.

Figure 7 and the inset to Figure 7 show σ_xy_ and σ_xx_ , together with fits to Equations 1 and 2 over limited ranges for Sample C, respectively. From the fits, it is possible to determine the respective renormalized mobilites as a function of temperature as shown in Figure 8a for Sample C and in Figure 8b for Sample A. The renormalized mobility calculated using Equation 1 is only slightly larger than that using Equation 2. It may be possible that different mobilities should be taken into account to understand the direct I-QH transition [37,43,45].

## Conclusions

In conclusion, the authors have presented studies on three completely different electron systems. In these three samples, the nanoscaled scatterers, in close proximity of the 2DES, provide necessary disorder for observing the direct I-QH transition. In these studies, it has been shown that the crossover from localization to Landau quantization actually covers a wide range of magnetic field. Moreover, the observed direct I-QH transition is not necessarily linked with Landau quantization as no resistance oscillations are observed even up to a magnetic field 4 *T *higher than the crossing field. Most importantly, the onset of strong localization which gives rise to the formation of quantum Hall state does not correspond to the direct I-QH transition. All these three pieces of experimental evidence show that a 2DES in the vicinity of nanoscaled scatterers is an ideal playground for studying the direct I-QH transition. Furthermore, in order to obtain a thorough understanding of the underlying physics of the direct I-QH transition, modifications of Huckestein's model [20] must be made.

## Abbreviations

I-QH: insulator-quantum Hall; SdH: Shubnikov-de Haas; 2DESs: two-dimensional electron systems.

## Competing interests

The authors declare that they have no competing interests.

## Authors' contributions

CTL, GHK and YHC coordinated the measurements on Sample A. CTL coordinated the measurements on Sample B. KYC performed the measurements on Sample B. JCC and YL coordinated the measurements on Sample C undertaken in Taiwan. YO and NA coordinated early measurements on Sample C in Japan. CTL, STL and CFH drafted the manuscript. LHL, YTW and DLS performed measurements on Sample C. SDL and DAR grew the MBE wafers. All authors read and approved the final manuscript.
